# Recurrence of Pemphigus Vulgaris Under Nivolumab Therapy

**DOI:** 10.3389/fmed.2019.00262

**Published:** 2019-11-12

**Authors:** Sebastian Krammer, Christian Krammer, Suzanna Salzer, Işin Sinem Bağci, Lars E. French, Daniela Hartmann

**Affiliations:** ^1^Department of Dermatology and Allergology, University Hospital, LMU Munich, Munich, Germany; ^2^Department of Dermatology, Municipal Hospital of Munich, Munich, Germany

**Keywords:** pemphigus vulgaris, nivolumab, PD-1, checkpoint inhibitors, autoimmune disease, autoimmune vesiculobullous disease, NSCLC, confocal microscopy

## Abstract

For many types of cancer, immune checkpoint inhibitors have proven to be a highly effective treatment. The monoclonal anti-PD-1 antibody nivolumab stimulates the immune system and is one of the newest treatment options for non-small cell lung cancer. In doing so, immune checkpoint inhibitors can trigger many skin lesions that have not yet been completely investigated in their entirety. In this case report, pemphigus vulgaris is identified as a potential adverse event that occurs under the treatment with nivolumab. In addition to the standard methods, we examined our patient's samples with *ex vivo* confocal laser scanning microscopy. This is a new and innovative diagnostic method that uses vertical scanning to provide fast, high-resolution imaging of freshly excised tissue, even using fluorescently labeled antibodies.

## Background

With the increasing use of immune checkpoint inhibitors (ICIs) the frequency of immune-related adverse events (irAEs) is steadily increasing. The most common immune-mediated adverse events appear on the skin (46–62%), followed by immune-mediated colitis (22–48%) and immune-mediated hepatitis (7–33%) (1). Nivolumab is an indirect antitumor agent from the monoclonal antibody group used to treat a variety of cancer types. It binds to the PD-1 receptor on T cells and inhibits the interaction with the ligands PD-L1 and PD-L2 on cancer cells. This enhances the body's immune response against the tumor (2–4). Based on this mode of action, diverse immune reactions are expected as adverse events. The most common possible adverse events associated with nivolumab include fatigue, skin rash, pruritus, immune-mediated pneumonitis, colitis/diarrhea, nausea, pyrexia and thyroid dysfunction (5–7). The exact underlying mechanism is still not fully understood, but it is postulated to be largely a T cell mediated reaction (4, 7–9).

This case report identifies pemphigus vulgaris (PV) as a potential adverse event developed under nivolumab therapy. To our knowledge, there is no report of a typical PV under nivolumab therapy to date. One case of an atypical pemphigus developed in a patient with urothelial carcinoma treated with nivolumab was reported so far (10).

Standard diagnostic evaluations of pemphigus vulgaris include direct immunofluorescence (DIF), histopathological examination, and immuno-serological examinations (11). An innovative examination method, the *ex vivo* confocal laser scanning microscopy (CLSM), offers an ultra-rapid vertical scanning of skin samples with a resolution close to conventional histology and a possibility to apply immunofluorescent dyes and specific antibodies (12–15). In addition, the *ex vivo* CLSM is faster than using DIF, which was previously established as the gold standard when performing immunostaining (14). We present the *ex vivo* CLSM examination results in our patient as a possible alternative to DIF (**Figures 2E–H**).

## Case Presentation

**Figure 1:**
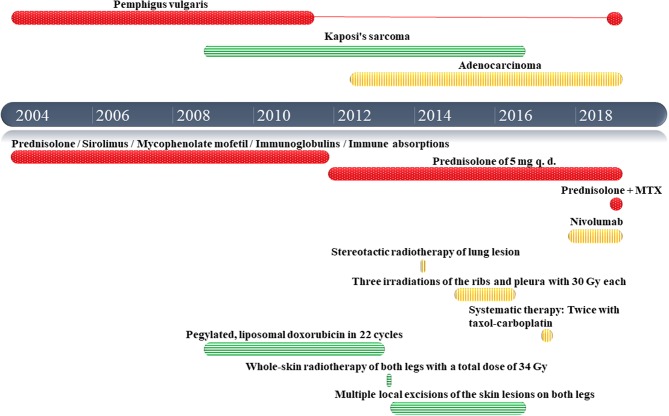
Timeline of the patient's diagnoses and treatments (in red information related to pemphigus vulgaris, in green information related to kaposi's sarcoma and in yellow information related to the lung adenocarcinoma).

In November 2018 an 85-year-old Caucasian man presented with lesions involving the skin on the entire integument and oral mucosa. The lesions developed 3 weeks prior to presentation. In 2004, the patient was diagnosed with PV for the first time. In addition, the patient's medical history included cutaneous Kaposi's sarcoma of the lower extremity diagnosed in 2008 and adenocarcinoma in the right upper lobe of the lung, TNM classification T3a N0 M1a, diagnosed in 2012. Figure 1 shows a timeline of the patient's diagnoses and treatments.

**Figure 2:**
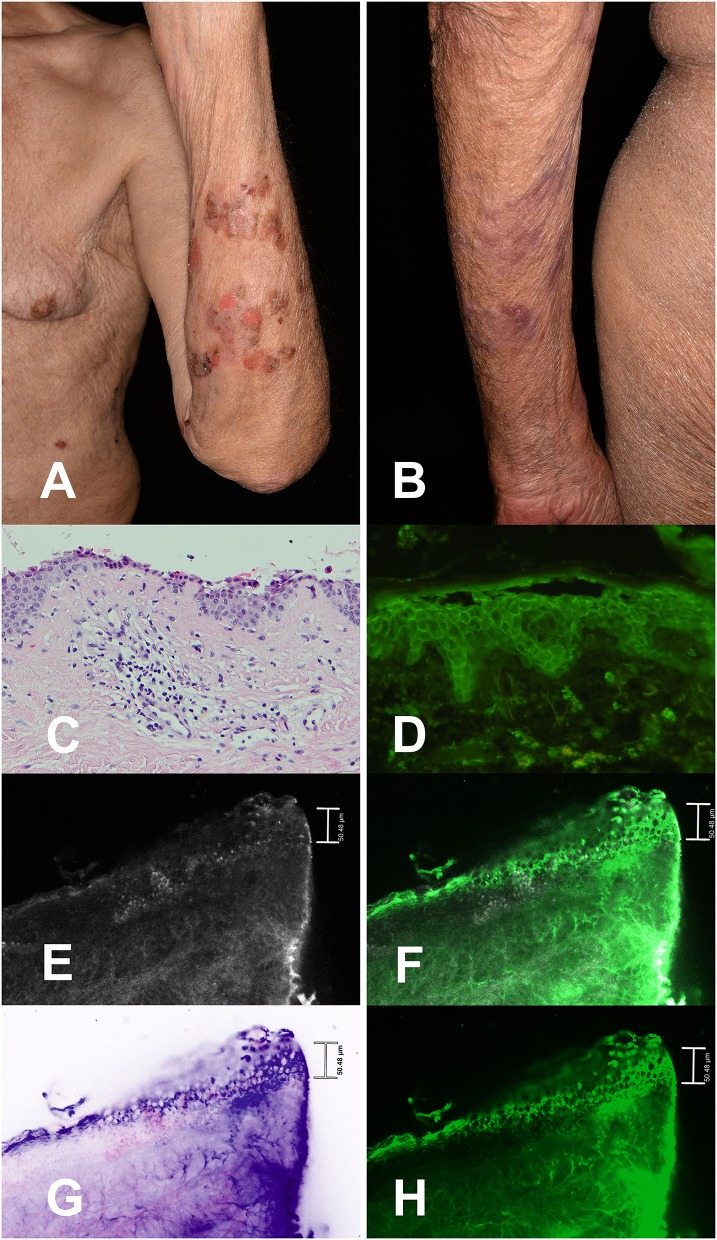
Pemphigus vulgaris lesions on the left forearm of a patient with lung adenocarcinoma treated with nivolumab showing multiple erosions and hemorrhagic crusts. Fresh blisters are not present anymore **(A)**. Skin condition of the patient 4 weeks later under therapy with prednisolone 20 mg daily and methotrexate 10 mg weekly as well as topical therapy **(B)**. Detailed image of lesional epidermis, dermo-epidermal junction (DEJ) and dermis in histology (200 ×) showing a suprabasal acantholysis as well as perivascular infiltrates of lymphocytes, histiocytes, eosinophilic, and neutrophilic granulocytes in the upper dermis. The upper part of the epidermis is focally missing due to the intralesional punch biopsy **(C)**. Perilesional direct immunofluorescence microscopy with FITC-labeled anti-human-IgG-antibodies (250 ×) showing intercellular deposition of IgG in the epidermis **(D)**. *Ex vivo* confocal laser scanning microscopy of perilesional biopsy specimen with IgG-antibodies **(E–H)** showing histomorphological details as well as specific intercellular binding of the IgG-antibodies mainly in the lower half of the epithelium in different imaging modes: Reflectance mode **(E)**, overlay of reflectance and fluorescence mode **(F)**, digital staining mode **(G)**, and fluorescence mode **(H)**.

Table 1 shows additional health information including his long-standing medication. Furthermore, Figure 2A shows multiple erosions and hemorrhagic crusts of the patient's PV lesions on his left forearm on admission day.

**Table 1:** Additional patient's health information.

**Health condition**	**Date of onset**
Type 2 diabetes mellitus	~2000
Stage II chronic renal failure stage II	~2013
3-vessel coronary heart disease, non-ST elevation myocardial infarction	01/2013
Myocardial infarction with double stent implantation	1999
Status post ventricular tachycardia	2013
Status post electrical cardioversion with moderate ejection fraction	unknown
Arterial hypertension	~1980
Status post herpes zoster L1-L3	08/2008
Status post nicotine abuse until 1995 (25 pack years)	1970
**Long-standing medication**	
**Medication and dose**	**Mode of administration**
Prednisolone 5 mg	1-0-0 p.o.
Pantoprazole 40 mg	1-0-0 p.o.
Aspirine 100 mg	1-0-0 p.o.
Amiodarone 200 mg	0-1-0 p.o.
Bisoprolol 2.5 mg	0-1-0 p.o.
Simvastatin 20 mg	0-0-1 p.o.
Enalapril 10 mg	1-0-0 p.o.

## Clinical and Laboratory Findings

Upon physical examination multiple superficial skin erosions and several blisters of up to 2 cm diameter were seen. Additionally, discrete erosions of the oral mucosa were noted. The Nikolsky's sign I (direct) and II (indirect) were both positive. The dermatohistopathologic report showed a suprabasal clefting, which turned into a blister. The blister lumen was filled with fibrin, acantholytic cells, eosinophils and neutrophils. The DIF examination revealed blister formation in the basal epidermis as well as intercellular deposits of FITC-labeled anti-IgG-antibodies in the entire epidermis but not in the basement membrane zone. In conclusion the dermatohistopathology report was consistent with PV, as was DIF. The indirect IF (IIF) was pemphigus positive and pemphigoid negative, desmoglein 1 (129,3 U/ml; reference positive >20 U/ml) and 3 (64,7 U/ml; reference positive >20 U/ml) positive Elisa, monkey IgG titer and rabbit IgG titer with 1:10 positive, paraneoplastic pemphigus laboratory testing negative (negative rat urinary bladder and negative monkey urinary bladder). The histopathological and *ex vivo* confocal morphology of the patient's skin is presented in Figures 2C–H.

## Therapy and Course of PV

After his initial PV diagnosis in 2004, the patient was treated with prednisolone, sirolimus, mycophenolate mofetil, immunoglobulins, and immune absorptions until November 2011. By 2018, the patient's PV was in remission without blister formation under a dose of prednisolone of 5 mg orally daily.

Upon relapse following Nivolumab therapy in November 2018, the patient was topically treated with betamethasone/triclosan cream. The topical prednisolone dose was gradually reduced in the course of 3 weeks.

Moreover, he received a systemic therapy with prednisolone 60 mg orally daily and methotrexate (MTX) 7.5 mg s.c. once a week including folic acid substitution.

The gradual reduction of the prednisolone dose to the initial one of 5 mg daily and simultaneously administration of an increased dose of MTX (up to 10 mg once per week) followed.

## Therapy and Course of Lung Adenocarcinoma

Therapy with nivolumab was started in November 2017. Nineteen cycles of immunotherapy with nivolumab (200 mg nivolumab intravenously, initially every two weeks, later 240 mg every 4 weeks) were completed before his inpatient stay in November 2018.

The patient showed a good clinical response under nivolumab therapy with reduced thoracic pain and less dyspnea. Carcinoembryonic antigen (CEA)-values decreased accordingly.

## Follow-Up

Complete healing of the skin occurred within 8 weeks after the initiation of the above-mentioned dermatological therapy in November 2018. The patient continued the nivolumab therapy and has not developed any new skin lesions within the last 6 months. Figure 2B shows the clinical appearance of the patient's PV lesions on the left forearm 4 weeks after therapy.

## Discussion

It is expected that ICIs will become a standard as first-line treatment, either as monotherapy or in combination with chemotherapy, for advanced or metastatic non-small-cell lung cancer (16). We describe the recurrence of pre-existing PV under anti-PD-1 therapy in a patient with lung adenocarcinoma. Nivolumab enhances the body's immune response to the tumor, but can also cause an immune response in other organs (17). We hypothesize that nivolumab might have triggered the recurrence of typical PV in our patient. Normally the average time to onset of irAEs ranges from 5 weeks for skin-related effects to 15 weeks for renal effects (18). Giulia et al. have described in their retrospective study about anti-PD-1 inhibitors in the treatment of non-small-cell lung cancer in patients with pre-existing autoimmune disease that the onset of flare symptoms was highly variable, ranging from 1 to 260 days after starting the immunotherapy (19). Therefore, we conclude that pemphigus vulgaris can also recur after a longer time period as mentioned above.

In the literature, only one case of an atypical pemphigus developed in a patient with no history of autoimmune disease upon receiving nivolumab for the treatment of urothelial carcinoma has been reported so far (10). The reporting of autoimmune related adverse events is important, considering that most patients with pre-existing autoimmune diseases were excluded from the large clinical trials with immune checkpoint inhibitors due to the association between pre-existing autoimmune diseases and the risk of immune related adverse events (20). Nevertheless, immunotherapy should not necessarily be ruled out in patients with a pre-existing autoimmune disease. For example, Maeda et. al. have described the use of immune checkpoint inhibitors in a patient with pre-existing pemphigus foliaceus without any exacerbation of the autoimmune disease (21).

Besides, there are case reports of patients who have developed pemphigus after taking enalapril (22–24). In our case, the patient has been taking enalapril for several years (>5 years). We therefore saw no direct relationship between the PV and the use of the patients's long-term medication enalapril. However, it cannot be entirely ruled out that the combination of enalapril and nivolumab had a synergistic effect on the relapse of PV.

Moreover, it should be noted that there is further evidence in the literature that acantholysis in pemphigus vulgaris may be not only due to a stereotactic disorder of cell adhesion molecules such as desmoglein 3 or desmoglein 1 by the autoantibodies, but also due to an autoantibody-independent process with altered keratinocyte intracellular signaling. Hitherto unknown intracellular signaling cascades, which are stimulated by autoantibody binding, may lead to an increase of intracellular calcium and in addition to the phosphorylation of desmoglein 3 as well as to the decoupling of desmoglein 3-plakoglobulin complex (25–27).

Furthermore, in a recent case series of 56 patients with pre-existing autoimmune diseases receiving anti-PD-1 therapy, 55 % developed a flare of underlying autoimmune disease or an immune related adverse event, although most events were mild (19).

In March 2019, Kehl et al. published the biggest observational study thus far including 4,438 patients treated with immune checkpoint inhibitors. Four hundred and sixty two patients had pre-existing autoimmune diseases. No significance was shown with all-cause hospitalizations, only a modestly increased rate of hospitalization with a diagnosis consistent with immune related adverse events was observed (20).

Systemic glucocorticoids have long been the standard first-line treatment in PV but are associated with considerable morbidity. Several studies in patients with PV found that MTX is a useful and well-tolerated therapy with a steroid-sparing effect (28–30). We assumed that MTX would be best tolerated by our patient compared to all other standard immunosuppressants (systemic corticosteroids, mycophenolate mofetil, azathioprine, cyclophosphamide, dapsone and cyclosporine) and that it would be associated with the lowest risk of tumor progression of the patient's lung adenocarcinoma (28). The patient tolerated the therapy well and remission could be reached.

Typically, the therapy of nivolumab irAEs includes glucocorticoid administration and subsequent tapering. In case of glucocorticoid-refractory adverse events without improvement of symptoms, the immunosuppression should be escalated immediately. In the absence of a response, the exclusion of infections is recommended (1).

Patients should be informed that in case of a ICIs' interruption due to adverse events, the efficacy may not necessarily be reduced. This prevents patients from underreporting possible adverse events due to their fear of discontinuation of the therapy (1). Moreover, long-term follow-ups are essential to allow for a more detailed characterization of skin adverse events associated with PD-1 inhibitors.

*Ex vivo* CLSM is a relatively new diagnostic method that has been established in dermatology in the last few years. Only recently, the possibility of using FITC (fluorescein isothiocyanate)-labeled antibodies in *ex vivo* CLSM was firstly described in two pilot studies (12, 14). The images presented in our case present one of the first applications of FITC-labeled antibodies in *ex vivo* confocal CLSM. In future, even higher quality images in comparison to DIF can be expected, since the *ex vivo* CLSM method and its devices are continuously being improved.

Further it would also be possible to monitor the therapy additionally by *in vivo* CLSM. Compared to *ex-vivo* CLSM continuous non-invasive monitoring is possible. Thus, more conclusions regarding our presumed trigger nivolumab and pemphigus vulgaris could be investigated.

## Conclusion

Anti-PD-1 monoclonal antibodies show promising effects in the treatment of diverse cancer types and lead to improved outcomes. The increased use of ICIs may lead to an increasing number of serious autoimmune irAEs reported (31). Under nivolumab therapy the occurrence of PV seems to be a rare, but potentially serious dermatological side effect. Given our patient's good clinical response to the selected therapy, we decided, in consultation with the responsible oncologists, to continue the immunotherapy. Nonetheless, discontinuation of anti–PD-1 therapy may sometimes be required.

Precise and structured documentation of side effects is essential in order to detect them in specific patient populations and at an early stage. This could contribute to the identification of risk factors for the development of known side effects (1).

Prompt referral to a specialist in case of suspicious signs is strongly recommended. Interdisciplinary cooperation is crucial for optimal patient management and treatment of immune-related side effects (1, 31).

## Ethics Statement

Written informed consent was obtained from the patient for the publication of this case report.

## Disclosure

The Prototype Vivascope 2500M-G4^®^ device was provided by Mavig GmbH.

## Author Contributions

SK and CK: concept, writing, and diagnostics. SS: proofreading and language editing. IB: diagnostic support. LF and DH: supervision, proofreading, and editing.

### Conflict of Interest

The authors declare that the research was conducted in the absence of any commercial or financial relationships that could be construed as a potential conflict of interest.
